# CAREERS IN ACADEMIC GEROPSYCHOLOGY: STRENGTHENING THE PIPELINE

**DOI:** 10.1093/geroni/igz038.2224

**Published:** 2019-11-08

**Authors:** Jessica Strong, Matthew Wynn, Jennifer Moye, Brian Carpenter

**Affiliations:** 1 VA Boston Healthcare System, Boston, Massachusetts, United States; 2 Washington University St. Louis, St. Louis, Missouri, United States

## Abstract

The shortage of clinical geropsychologists is further imperiled by the shortage of geropsychologists entering academia. The current study analyzed data collected during the implementation of a national webinar series (Advancing your Confidence as an Educator in Geriatrics and Gerontology). The development and implementation of the series has been previously reported. In the current study, we report on n=66 psychologists’ and psychology trainees’ responses to academia. Results included quantitative ratings of feelings towards imaging oneself in an academic role. Feelings rated included Excitement (41%), Intimidated (18%), Overwhelmed (30%), and Enthusiastic (30%), among others. These results were triangulated with open-ended qualitative responses on the frustrations and rewards of being an educator. Results are discussed in the context of the geropsychology pipeline shortage and implications for the future of clinical geropsychology. Suggestions for how to remedy this concern will be discussed.

